# Electroacupuncture at LI11 promotes jejunal motility via the parasympathetic pathway

**DOI:** 10.1186/s12906-017-1826-9

**Published:** 2017-06-21

**Authors:** Xuanming Hu, Mengqian Yuan, Yin Yin, Yidan Wang, Yuqin Li, Na Zhang, Xueyi Sun, Zhi Yu, Bin Xu

**Affiliations:** Key Laboratory of Integrated Acupuncture and Drugs Constructed, Nanjing University of Chinese Medicine, Ministry of Education, Nanjing, 210023 China

**Keywords:** Electroacupuncture (EA), LI11, Jejunal motility, Parasympathetic pathway, Sympathetic pathway

## Abstract

**Background:**

Gastrointestinal motility disorder has been demonstrated to be regulated by acupuncture treatment. The mechanisms underlying the effects of acupuncture stimulation of abdominal and lower limb acupoints on gastrointestinal motility have been thoroughly studied; however, the physiology underlying the effects of acupuncture on the forelimbs to mediate gastrointestinal motility requires further exploration. The aim of this study was to determine whether electroacupuncture (EA) at LI11 promotes jejunal motility, whether the parasympathetic pathway participates in this effect, and if so, which somatic afferent nerve fibres are involved.

**Methods:**

A manometric balloon was used to observe jejunal motility. The effects and mechanisms of EA at LI11 were explored in male Sprague-Dawley rats with or without drug administration (propranolol, clenbuterol, acetylcholine, and atropine) and with or without vagotomy. Three types of male mice (β_1_β_2_ receptor-knockout [β_1_β_2_^−/−^] mice, M_2_M_3_ receptor-knockout [M_2_M_3_^−/−^] mice and wild-type [WT] mice) were also studied by using different EA intensities (1, 2, 4, 6, and 8 mA). A total of 72 rats and 56 mice were included in the study.

**Results:**

EA at LI11 increased the contractile amplitude of jejunal motility in the majority of both rats and mice. However, EA at LI11 did not enhance jejunal motility in rats administered atropine, rats that underwent vagotomy, and M_2_M_3_^−‍‍/−^ mice (at all intensities). In WT mice, EA at LI11 significantly increased jejunal motility at all intensities except 1 mA, and a plateau was reached at intensities greater than 4 mA.

**Conclusion:**

Our results suggest that EA at LI11 promotes jejunal motility primarily by exciting the parasympathetic pathway, and that Aδ-fibres and C-fibres may play important roles in the process.

**Electronic supplementary material:**

The online version of this article (doi:10.1186/s12906-017-1826-9) contains supplementary material, which is available to authorized users.

## Background

Gastrointestinal motility disorder consists of multiple clinical symptoms and occurs in numerous diseases, including diarrhoea, constipation and irritable bowel syndrome (IBS) [1, 2]. Functional bowel disorders have become a common research focus in recent decades, owing to the rapid advances in neurogastroenterology and the development of many new methods to study gastrointestinal motility [3].

An important role of the somatic-autonomic reflex in gastrointestinal motility has been demonstrated, whereas the autonomic nervous system has been reported to be highly involved in modulating visceral organ function [4–8]. Further studies have shown that small bowel contractile activity is under nervous system control via parasympathetic excitatory and sympathetic inhibitory nerve fibres directly act on smooth muscle cells [9, 10].

Many studies have provided reliable evidence of the effectiveness of acupuncture therapy for treating gastrointestinal motility disorder, primarily through autonomic nerve reflexes [4, 11, 12]. In animal models, acupuncture stimulation of abdominal skin inhibits gastrointestinal motility primarily by exciting sympathetic pathways [13], whereas acupuncture at hind paws enhances gastric motility mainly via parasympathetic pathways [14, 15]. Electroacupuncture (EA) is a variant of manual acupuncture (MA) in which electrical stimulation is applied through needles. Owing to the ease of modulating its stimulation frequency and intensity, EA is recognized as a quantifiable treatment and is widely used in clinical and experimental research [16]. For this reason, we chose EA as the stimulation method in this study.

Previous research has focused on stimulation of the abdomen or the hind paws, whereas few studies have used the forepaws. LI11 (Quchi), located at the midpoint between the lateral end of the transverse cubital crease and the lateral humeral epicondyle [14], is traditionally used to regulate gastrointestinal motility, because there sufficient supporting clinical and experimental evidence of its efficacy [17–26]. However, in contrast to other acupoints for regulating gastrointestinal motility, such as ST36 (Zusanli) and ST25 (Tianshu), the more evidence from basic research regarding the use of LI11 in acupuncture is needed.

We hypothesized that the immediate effect of EA stimulation at LI11 would be promoting jejunal motility, primarily by exciting parasympathetic pathways and inhibiting sympathetic pathways.

## Methods

### Animals

Male Sprague-Dawley rats (180–230 g; Model Animal Research Center of Nanjing Medical University, China) and male mice (22–28 g), including β_1_β_2_^**−/−**^ mice (B6.129X1-β_1_β_2_^tm1Jul/NJU^, J003810; donated by the Jackson Laboratory, USA), M_2_M_3_^**−/−**^ mice (B6.129X1-M_2_M_3_^tm1Jul/NJU^, D0407; from Kumamoto University, Japan), and wild-type counterparts (WT mice; purchased from the Model Animal Research Center of Nanjing University, China) were used in this study. Gene knockout in mice (β_1_β_2_^**−/−**^ mice and M_2_M_3_^**−/−**^ mice) was verified with PCR, and their genetic background is shown in the Additional file 1.

The animals were housed under a temperature of 22 °C and a relative humidity of 40%–60% at the SPF Experimental Animal Center, Nanjing University of TCM. They were housed under a 12 h/12 h light/dark cycle and were given free access to food and water. Animals underwent an adaptation period for seven days before inclusion in experiments. All experimental manipulations were undertaken in accordance with the Principles of Laboratory Animal Care and the Guide for the Care and Use of Laboratory Animals, published by the National Science Council, China.

### Drugs

All animals in the study were anaesthetized with urethane (U2500; Sigma, St. Louis, MO, USA). The beta adrenoceptor agonist clenbuterol hydrochloride (clen; C5423; Sigma, St. Louis, MO, USA) and the beta adrenoceptor antagonist propranolol hydrochloride (prop; P0084; Sigma) were administered to the rats. Next, the muscarinic receptor antagonist atropine hydrochloride (atropine; A6625; Sigma) and the agonist acetylcholine hydrochloride (ACh; A0132; Sigma) were administered. Penicillin antibiotic (B151226; Shandong Lukang Pharmaceutical Co., Ltd., Jining, China) was administered after surgery. The concentration, doses, and administration methods were 1) urethane: 20%, 8 mL/kg for rats and 5 mL/kg for mice, I.P.; 2) clenbuterol: 0.2%, maintenance dose of 80 μL/kg/min, intravenous (I.V.); 3) propranolol: 0.4%, initial dose of 1.0 mL/kg and maintenance dose of 40 μL/kg/min, I.V.; 4) acetylcholine: 0.1%, maintenance dose of 20 μL/kg/min, I.V.; 5) atropine: 0.2%, initial dose of 0.8 mL/kg and maintenance dose of 40 μL/kg/min, I.V.; and 6) penicillin: 8 × 10^5^ IU dissolved in 2 mL saline, 0.4 mL/d per rat, intramuscular (I.M.).

### Recording of the jejunal motility

All animals in the study were fasted for 12 h and given free access to water prior to anaesthetization with urethane. After supplementary anaesthesia, the rats in the drug-administered groups underwent endotracheal intubation to maintain the respiratory tract unobstructed and the left internal jugular venous catheter accessible for drug administration. A small incision (length: 5–8 mm in rats, 2–3 mm in mice) was made below the xiphoid, and a small balloon (diameter: approximately 2 mm) made of flexible rubber was inserted into the jejunum to approximately 3–5 cm (rats) or 0.8–1.2 cm (mice) distal to the duodenal suspensory ligament. The balloon, which was connected to a polyethylene tube (length: 10 cm), was then filled with 0.05–0.1 mL warm water to maintain the jejunal baseline pressure at approximately 0.3 kPa. The jejunal pressure was recorded with a transducer (YPJ01; Chengdu Instrument Factory, China) through the balloon and tube. The signal was collected with a biological signal-sampling system (RM6240; Chengdu Instrument Factory) for further analysis. The baseline pressure was maintained at 0.28–0.32 kPa. During the experiment, an electric heating board was used to maintain the temperature of the animals at 37 ± 0.5 °C.

### EA stimulation

LI11 (Quchi) is located midway between the lateral end of the transverse cubital crease and the lateral humeral epicondyle. A pair of stainless steel acupuncture needles (diameter: 0.3 mm) were inserted to a depth of 3 mm into the muscle layer at the right LI11. The needles were connected to a Han EA therapeutic stimulator (LH_4_02A; Beijing Huawei Industrial Development Corporation, China). The frequency of EA was set at 2/15 Hz for each stimulation.

### Vagotomy

The animals were anaesthetized with urethane, and a small incision was made in the midline of the abdomen. Animals then underwent bilateral vagotomy by dissection of the ventral and dorsal branches of the vagus nerve. The animals in the sham control group underwent the same procedure but without vagus nerve dissection. After suturing, the animals received penicillin antibiotic shots (0.2 mL each rat per day, I.M.) and were allowed three days to recover before the experimental session. Control group animals did not undergo any treatment before experiments. Twenty-four rats were used in this experiment.

### Study design

To verify our hypothesis, we sought to 1) demonstrate whether EA at LI11 could promote jejunal motility; 2) explore the effects of EA at LI11 on the sympathetic pathway and the parasympathetic pathway, and 3) explore which somatic afferent nerve fibres participate in the stimulation process associated with EA at LI11.

In the first experiment, we divided animals into four groups (rats with EA, rats without EA, mice with EA and mice without EA) and then recorded the jejunal pressure to describe the characteristics of jejunal motility.

In the second experiment, we first used drugs to excite or inhibit a specific pathway before EA stimulation. Then, we cut the gastro-vagal nerves of the rats to evaluate the role of the parasympathetic pathway. Lastly, β_1_β_2_ adrenoceptor double-knockout (β_1_β_2_^**−/−**^) mice, M_2_M_3_ muscarinic double-knockout (M_2_M_3_^**−/−**^) mice, and wild-type mice were used to further verify the results.

**Fig. 1:**
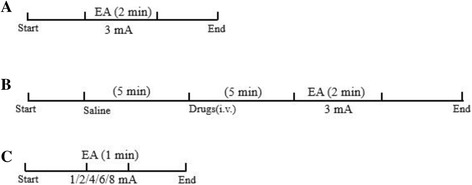
The experimental procedure. **a** Intervention timeline in rats stimulated by ordinary EA. **b** Intervention timeline in rats with drug (clenbuterol, propranolol, acetylcholine or atropine) administration. **c** Intervention timeline in three kinds of mice (wild-type, β_1_β_2_^−/−^, M_2_M_3_^−/−^) with different intensities of electroacupuncture (1, 2, 4, 6, and 8 mA)

In the last experiment, we divided animals into three groups (WT group, β_1_β_2_^**−/−**^ group and M_2_M_3_^**−/−**^ group) and used different intensities of EA to explore the mechanism by which somatic afferent nerve fibres participate in the stimulation process associated with EA at LI11. The procedure and details of the experiments are shown in Fig. 1. There were 8 animals per group in all experiments.

### Assessment

Jejunal pressure during EA (dur-EA) was compared with the jejunal pressure before EA was performed (pre-EA) [27]. The response was identified as being enhanced if the dur-EA pressure was >105% of the pre-EA pressure. The formula used to calculate the percentage change is given below (1).

1 $$ \mathrm{Percentage}\kern0.5em \mathrm{change}=\frac{\mathrm{dur}\hbox{-} \mathrm{EA}}{\mathrm{pre}\hbox{-} \mathrm{EA}}\times 100\% $$

### Statistical analysis

Results are expressed as the mean ± SEM (standard error of the mean). Data were analysed using SPSS 19.0 (IBM, Armonk, NY, USA) and GraphPad Prism 5.0 (GraphPad Software, La Jolla, CA, USA) software programs. Paired-sample *t* tests (within the group) and one-way ANOVA (between groups) were used for statistical analyses. *P* < 0.05 was considered statistically significant. The data curves for different intensities were fitted with eq. (2) (X: log of intensity; Y: response, increasing as X increases. Top and bottom: plateaus in the same units as Y. LogEC50: same log units as X).

2 $$ \mathrm{Y}=\mathrm{Bottom}+\frac{\left(\mathrm{Top}\hbox{-} \mathrm{Bottom}\right)}{1+10^{\mathrm{X}\hbox{-} \mathrm{LogEC}50}} $$

## Results

### Characteristics of jejunal motility of normal animals with or without EA at LI11

**Fig. 2:**
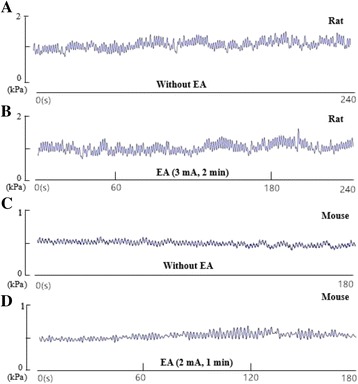
Changes in jejunal motility without or with EA at LI11. **a** A representative tracing of jejunal motility in a rat without any treatment. **b** A representative tracing of jejunal motility enhanced by EA at LI11 in a rat. **c** A representative tracing of jejunal motility in a mouse without any treatment. **d** A representative tracing of jejunal motility enhanced by EA at LI11 in a mouse. EA, electroacupuncture

After the injection of warm water into the balloon and stabilization of jejunal motility, we observed that the jejunal pressure was maintained in rats at approximately 0.8–1.0 kPa (Fig. 2a) and in mice at approximately 0.4–0.5 kPa, after injecting 0.05 mL warm water into the balloon (Fig. 2c). EA at LI11 induced increases in the contractile amplitude of jejunal motility in both mice and rats. In mice, the amplitude increased by approximately 0.05–0.3 kPa, and for rats, it increased by approximately 0.01–0.05 kPa (see Fig. 2b and d). The results suggested that EA at LI11 promotes jejunal motility.

### The role of the sympathetic pathway in the effect of EA at LI11

We used the beta adrenoceptor agonist clenbuterol and antagonist propranolol in rats and then performed EA at LI11 at an intensity of 3 mA. We divided rats into three groups: a control group (no administration), a clenbuterol group and a propranolol group (8 rats per group). Jejunum movement was markedly inhibited by the beta receptor agonist but was excited by propranolol.

**Fig. 3:**
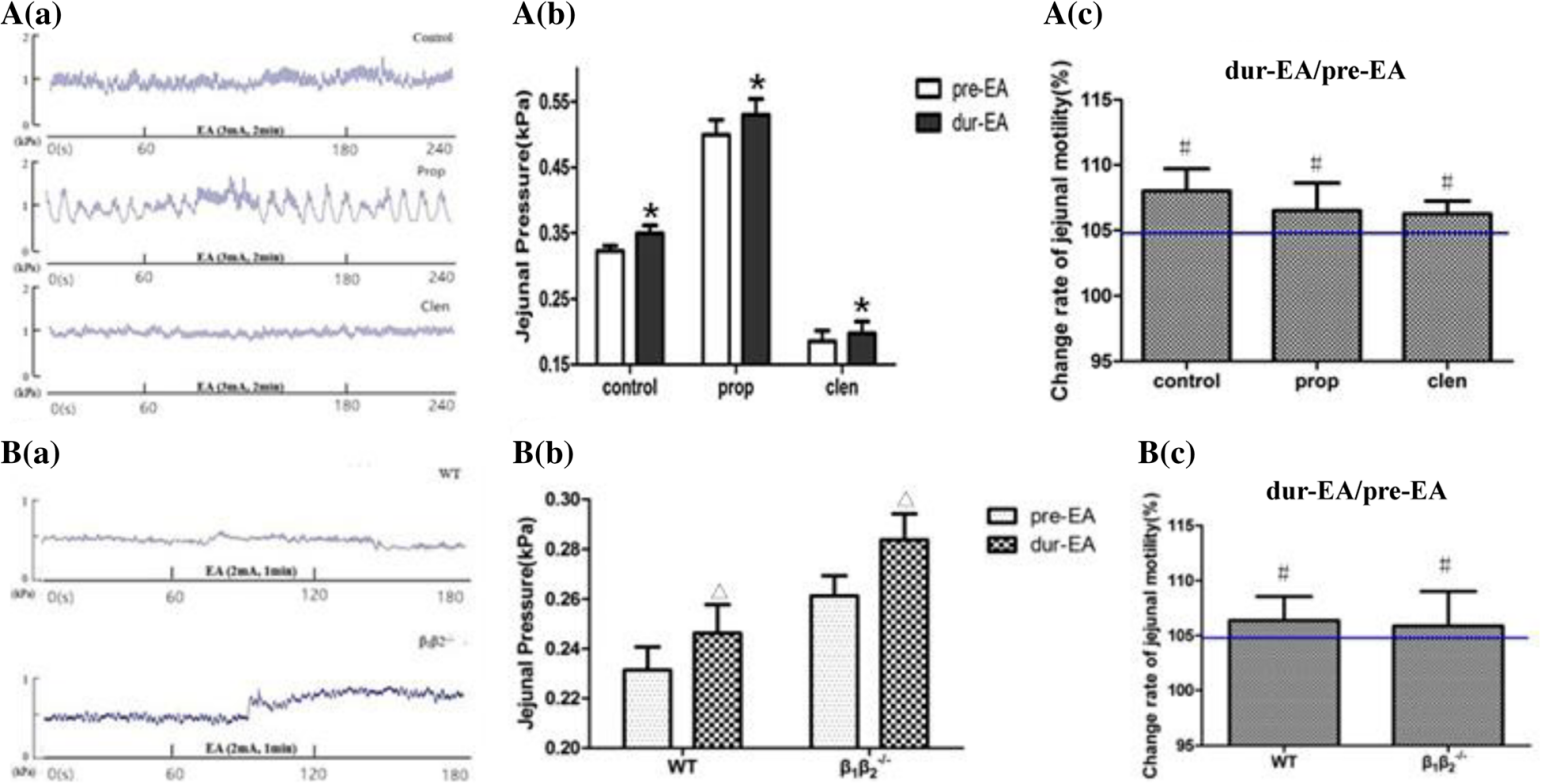
Jejunal motility in response to EA at LI11 under the administration of the sympathetic pathway. **Aa** Representative tracings of jejunal motility regulated by EA without and with the administration of propranolol and clenbuterol in rats. **Ab** Compared with the jejunal pressure before EA, changes were observed during EA in the control group, propranolol group and clenbuterol group. Propranolol promoted jejunal pressure significantly and clenbuterol led to the opposite effect, whereas EA at LI11 increased jejunal pressure in all three groups, which had statistical significance. Data are expressed as mean ± SEM (*n* = 8 rats per group at each time period). ^*^ *P* < 0.05 vs pre-EA, paired *t*-test. **Ac** Promotion percentages of jejunal pressure by EA in three rat groups are shown, and each change rate was above 105%. The promotion rate of EA was not significantly different among the groups. ^*#*^ *P* > 0.05 vs each group, One-way ANOVA. Data are expressed as mean ± SEM (*n* = 8 rats and mice). **Ba** Representative tracings of jejunal motility regulated by EA in WT mice and β_1_β_2_^−/−^mice. **Bb** Compared with the jejunal pressure before EA at the same intensity, changes were observed during EA in both the WT and β_1_β_2_^−/−^ groups. Thus, EA at LI11 increased jejunal pressure of both groups of mice significantly. Data are expressed as mean ± SEM (*n* = 8 mice per group at each time period). ^Δ^ *P* < 0.05 vs pre-EA, paired *t*-test. **Bc** Promotion percentages of jejunal pressure by EA in two mouse groups are shown, and each change rate was above 105%. The promotion rate of EA was not significantly different among the groups. ^*#*^ *P* > 0.05 vs each group, One-way ANOVA. Data are expressed as mean ± SEM (*n* = 8 rats and mice). EA, electroacupuncture; prop, propranolol; clen, clenbuterol. WT, wild-type

As shown in Fig. 3Ab and Ac, we found that EA induced excitement of jejunum movement in animals in all groups, corresponding to changes greater than 105%, whereas no significant differences were observed among groups. To further substantiate whether the sympathetic pathway was involved in the effect of EA at LI11, we applied EA stimulation of 2 mA to the LI11 of β_1_β_2_^**−/−**^ mice and found no significant differences between WT and β_1_β_2_^**−/−**^ mice (8 mice per group, Fig. 3Bb and Bc). These data suggested that the sympathetic pathway may not play a key role in the effect of EA at LI11.

### The role of the parasympathetic pathway in the effect of EA at LI11

Next, to determine whether the parasympathetic pathway was involved in the effect of EA at LI11 in regulating jejunal motility, we used the muscarinic receptor agonist ACh and antagonist atropine. We divided rats into three groups (control group, Ach group and atropine group) with 8 rats per group.

**Fig. 4:**
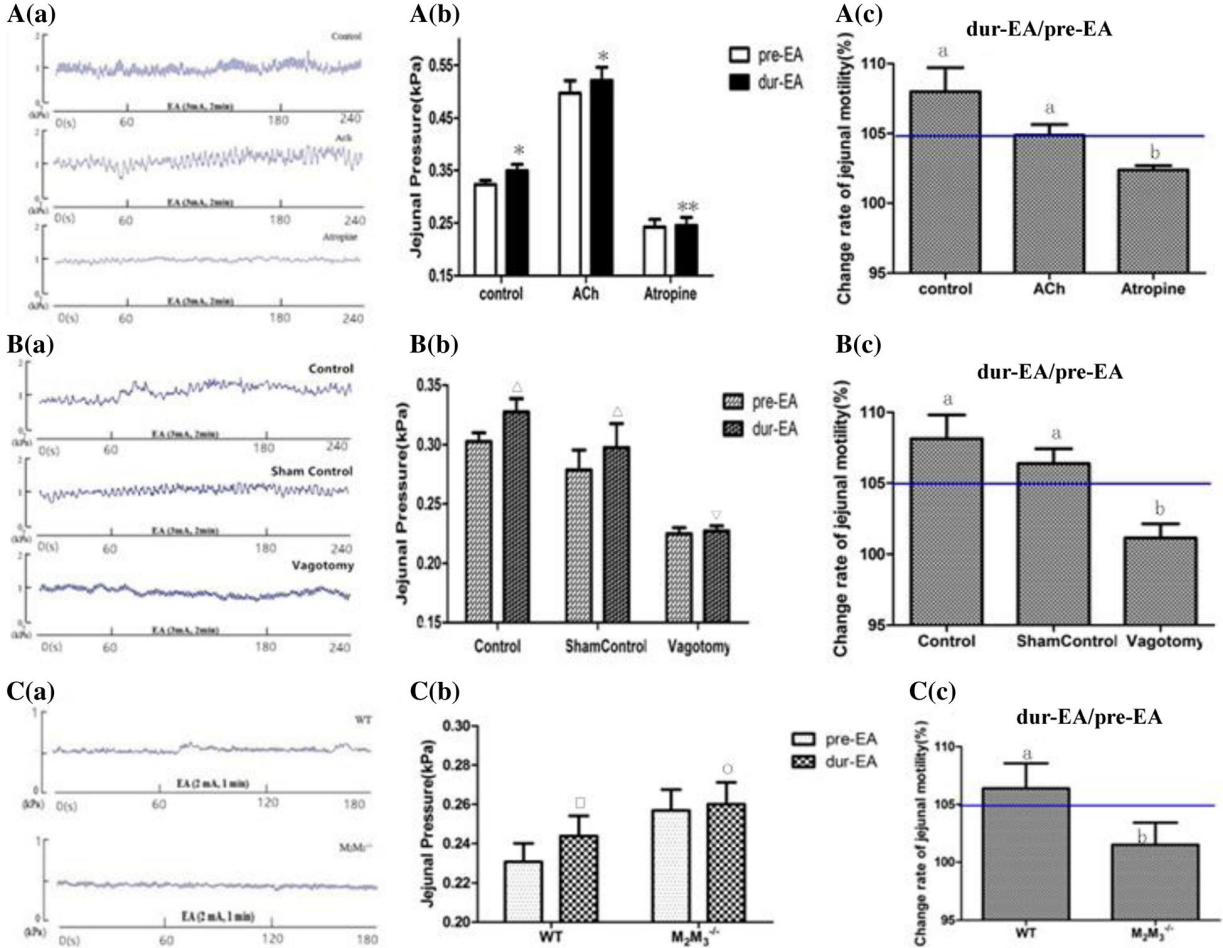
Jejunal motility in response to EA at LI11 under the administration of the parasympathetic pathway. **Aa** Representative tracings of jejunal motility regulated by EA, EA with the administration of ACh and atropine in rats. **Ab** Compared with the jejunal pressure before EA, changes were observed during EA in the control group and ACh group, but EA at LI11 failed to enhance jejunal pressure in the atropine group. Data are expressed as mean ± SEM (*n* = 8 rats per group at each period). ^*△*^ *P* < 0.05 vs pre-EA, ^#^ *P* > 0.05 vs pre-EA, paired *t*-test. **Ac** Promotion percentages of jejunal pressure from EA in the atropine group was below 105%, and were significantly lower than the other two groups. Data are expressed as mean ± SEM (*n* = 8 rats/mice). ^a^ *P* < 0.05 vs control group, Ach group, ^b^ *P* > 0.05 vs control group, Ach group, One-way ANOVA. **Ba** Representative tracings of jejunal motility regulated by EA, EA with the vagotomy, sham operation in rats. **Bb** Compared with the jejunal pressure before EA, changes were observed during EA in the control group and sham control group, but EA at LI11 failed to enhance jejunal pressure in the vagotomy group. Data are expressed as mean ± SEM (*n* = 8 rats per group at each period). △*P* < 0.05 vs pre-EA, ▽*P* > 0.05 vs pre-EA, paired *t*-test. **Bc** Promotion percentages of jejunal pressure from EA in the vagotomy group was below 105%, and were significantly lower than the other two groups. Data are expressed as mean ± SEM (*n* = 8 rats/mice). ^a^ *P* < 0.05 vs control group, Ach group, ^b^ *P* > 0.05 vs control group, Ach group, One-way ANOVA. **Ca** Representative tracings of jejunal motility regulated by EA in WT mice and M_2_M_3_^−/−^ mice. **Cb** Compared with the jejunal pressure before EA at the same intensity, the jejunal pressure in the WT group was enhanced with EA at LI11, but no impact was demonstrated in the M_2_M_3_^−/−^ group. Data are expressed as mean ± SEM (*n* = 8 mice per group at each period). ^*^ *P* < 0.05 vs pre-EA, ^○^ *P* > 0.05 vs pre-EA, paired *t*-test. **Cc** Promotion percentages of jejunal pressure from EA in the M_2_M_3_^−/−^ group were below 105%, and was significantly lower than the WT group. Data are expressed as mean ± SEM (*n* = 8 rats/mice). ^a^ *P* < 0.05 vs WT group, ^b^ *P* > 0.05 vs WT group, One-way ANOVA. EA, electroacupuncture; ACh, acetylcholine; WT, wild-type

The jejunal motility of the animals increased after Ach administration but decreased with atropine administration. We found that the internal pressure of the jejunum of rats in the Ach and control groups increased after EA stimulation. However, there were no obvious differences in jejunal motility between pre-EA and dur-EA after administration of atropine (from 0.24 ± 0.04 to 0.25 ± 0.04 in 8 rats, *P* > 0.05); hence, EA at LI11 did not enhance jejunal motility in the presence of atropine (Fig. 4Ab and Ac).

We then used rats with gastric vagotomy. We divided rats into three groups including a control group (no administration), a sham control group (subjected to the same procedure as the vagotomy group except for vagus nerve dissection) and a vagotomy group (8 rats per group). The experiment was performed three days after surgery. The results (see Fig. 4Ba, Bb and Bc) showed that movement of the jejunum was markedly inhibited and was not excited by EA stimulation when the bilateral gastro-vagal nerves were cut. The rats in the sham control group still exhibited enhanced jejunal motility after EA, as did the control group.

Furthermore, we used M_2_M_3_^**−/−**^ mice to determine the role of the parasympathetic pathway in the effect of EA at LI11.There were 8 mice in the WT group and in the M_2_M_3_^**−/−**^ group. The M_2_M_3_^**−/−**^ group also did not exhibit enhanced jejunal motility, thus further suggesting that the parasympathetic pathway may mediate the effect of EA at LI11 (see Fig. 4Cb and Cc). Together, these results demonstrated that the parasympathetic pathway plays an important role in the promotion effect of EA at LI11.

### Effects of EA at LI11 of different intensities on jejunal motility in mice

**Fig. 5:**
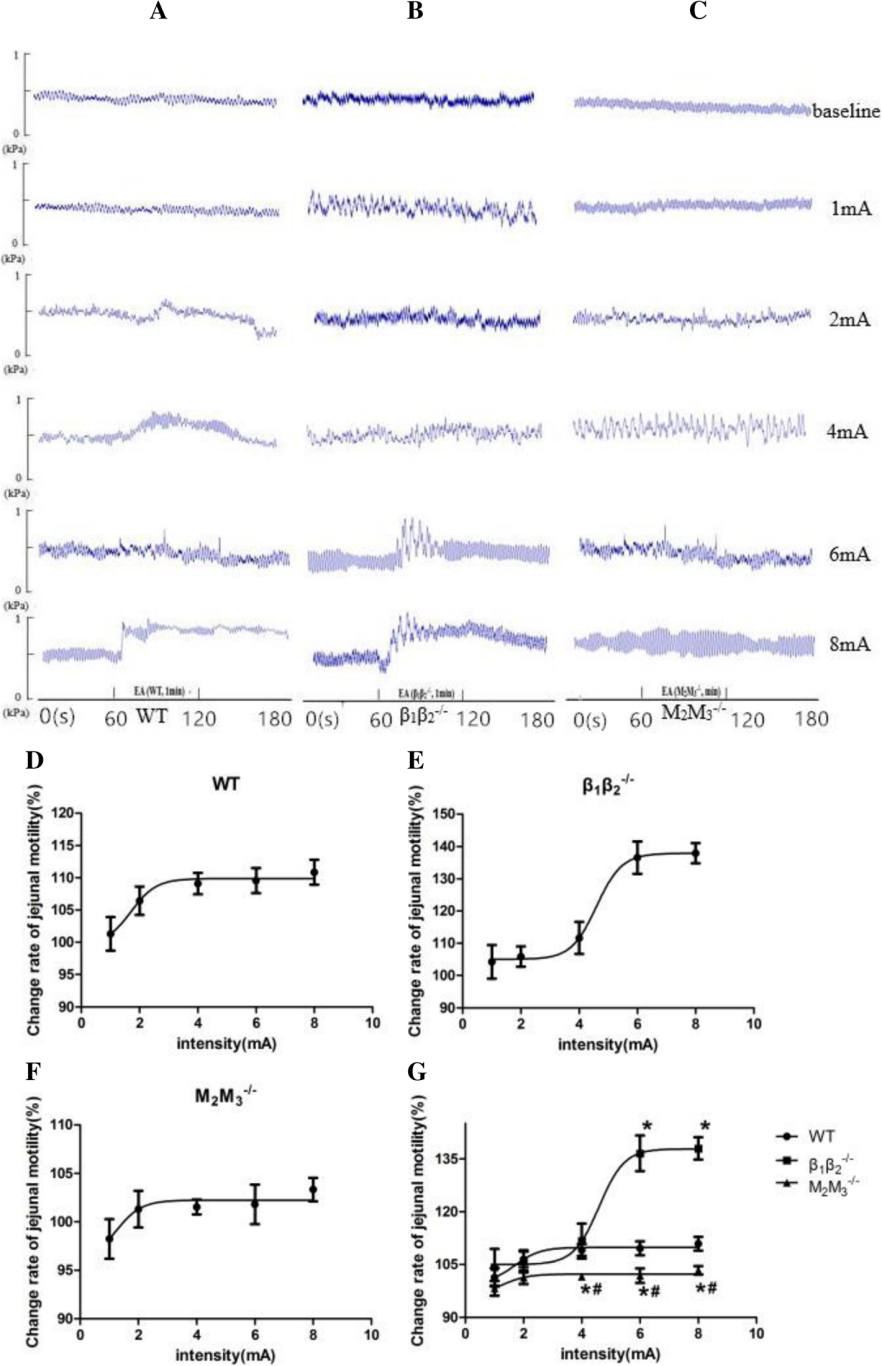
The effect of different intensities of EA at LI11 on jejunal motility in WT, β_1_β_2_^−/−^ and M_2_M_3_^−/−^ mice. **a**-**c** Representative tracings of jejunal motility with different intensities of EA in WT, β_1_β_2_^−/−^ and M_2_M_3_^−/−^ mice, respectively. **d**-**f** The fitting curves of the EA effect with different intensities on jejunal motility in WT, β_1_β_2_^−/−^ and M_2_M_3_^−/−^ mice, respectively, showing the relationships between different intensities of EA (1, 2, 4, 6, and 8 mA) and the rate of change rate in jejunal pressure. **g** The integrate fitting curve showed the enhanced effect of EA with different intensities on jejunal motility in three groups. Data are expressed as mean ± SEM (*n* = 8 mice). ^*^ *P* < 0.05 vs WT mice, ^#^ *P* < 0.05 vs β_1_β_2_^-/−^ mice, independent *t*-test. WT, wild-type

To gain further insight into the mechanism through which somatic afferent nerve fibres participate in the stimulation process associated with EA at LI11, different intensities of EA (1, 2, 4, 6, and 8 mA) were applied to three groups of mice (β_1_β_2_^**−/−**^, M_2_M_3_^**−/−**^ and WT mice). In the WT group, we observed that the promotion of jejunal motility by EA at LI11 with different intensities exceeded 105%, except for 1 mA stimulation (Fig. 5a and d). The logEC_50_ value of EA stimulation in WT mice was 1.71 ± 0.59 mA, and the promotion of jejunal motility increased in response to increasing EA intensity up to 4 mA. When the intensity exceeded 4 mA, a plateau in jejunal motility was reached (Fig. 5d).

The logEC_50_ value of EA stimulation in β_1_β_2_^**−/−**^ mice was 4.60 ± 0.41 mA, and the value in M_2_M_3_^**−/−**^ mice was 1.24 ± 1.77 mA. Compared with WT mice, the rate of change of jejunal pressure caused by EA at LI11 was significantly lower with intensities of 4 mA to 8 mA in M_2_ M_3_^−/−^ mice, and a similar trend was observed for 2 mA. However, the M_2_M_3_^−/−^ mice showed markedly different results as compared with those for other groups of mice subjected to EA of the same intensity, because EA at any intensity did not alter jejunal motility of these mice (Fig. 5c and f). No differences in the effects of EA were found between WT and β_1_β_2_^**−/−**^ mice stimulated at intensities of 1, 2, or 4 mA.

There was a sudden increase in the rate of change of jejunal motility when the EA intensity was increased to 4 mA. However, the motility subsequently reached a plateau and did not further change when the EA intensity was increased to 6 mA.

In general, these data further demonstrated that the parasympathetic pathway plays an important role in the jejunal mobility promotion effect of EA at LI11.

## Discussion

Gastrointestinal motility disorders occur in many gastrointestinal and other systemic diseases. Current drug therapy is not particularly effective at controlling the symptoms, and people with the disorder require long-term drug therapy. This therapy increases patients’ economic burden and is associated with increased risks of possible drug side effects [28–31]. Acupuncture has been reported to be an effective method of complementary and alternative medicine for the treatment of gastrointestinal motility disorders in numerous clinical studies [32–35]. LI11 (Quchi) is a useful acupoint on the arm that is often used to treat gastrointestinal diseases. It has been studied primarily for mediating gastrointestinal motility [17–26].

Along the gastrointestinal tract, normal peristalsis and homeostatic sensory and motor mechanisms are under the control of extrinsic parasympathetic and sympathetic pathways. By exploring the response to vagal stimulation of the jejunum and ileum in cats, Kewenter has found clear evidence that splanchnic sympathetic fibres exert an inhibitory effect on the ileum at the intramural ganglionic, but not directly on smooth muscle cells [36]. Although Sato et al. have found that abdominal stimulation inhibits gastrointestinal motility, stimulation of the hind paw enhances gastrointestinal motility in rats [4–6]. On the basis of these discoveries, Zhu Bing et al. [14, 15, 37–39] have studied the effects of acupuncture stimulation and its physiological mechanisms and have demonstrated that acupuncture stimulation at abdominal points inhibits gastrointestinal motility but that acupuncture at hind paw points enhances gastrointestinal motility. These authors have also defined “homotopic” and “heterotopic” acupoints as part of a new theoretical approach to interpret acupuncture effects and mechanisms. According to this approach, afferent innervation of a homotopic acupoint is in the same spinal cord segment from which the efferent branch innervates visceral organs, whereas afferent innervation is in a different segment for heterotopic acupoints. Acupuncture stimulation at homotopic points inhibits gastrointestinal motility via the sympathetic pathway; in contrast, acupuncture at heterotopic points enhances gastrointestinal motility via the parasympathetic pathway. Our previous study has shown that EA at ST25 inhibits gastric and jejunal motility via the sympathetic pathway; however, EA at ST37 excites gastric and jejunal motility via the parasympathetic pathway [13, 40]. These results support the “homotopic and heterotopic acupoints” theory. We then turned our focus to the gastrointestinal effect of acupuncture at forelimb points such as LI11 (Quchi) and its underlying mechanism, because LI11 is an effective acupoint in regulating gastrointestinal motility. In fact, many acupuncturists select this point for treating gastrointestinal motility disorders in clinical practice [17–22]. The experimental exploration of the effect of acupuncture at LI11 in regulating gastrointestinal motility has not received much attention in the past. Qin [14], however, has reported that acupuncture at LI11 (containing afferents from the C5 spinal dorsal horn) is a heterotopic acupoint to the jejunum (T9–12); this treatment increases the amplitude of peristalsis waves and enhances jejunal motility in normal rats as well as constipated and diarrheic rats. In our study, we used an antagonist and agonist for both adrenoceptors and muscarinic receptors to identify the roles of the sympathetic and parasympathetic pathways in normal rats. In addition, we used gene knock-out mice (β_1_β_2_^**−/−**^ mice and M_2_M_3_^**−/−**^ mice) for further verification. As shown in Fig. 2, we found a clear promotion effect of EA at LI11 on jejunal motility, in experiments using both rats and mice. Further explorations of the underlying neural mechanisms are shown in Figs. 3 and 4. There were three notable results of this study: 1) EA stimulation did not enhance jejunal motility in the presence of atropine in rats (Fig. 4a); 2) after vagotomy, the effect of EA on jejunal motility disappeared in rats (Fig. 4b); and 3) EA stimulation at LI11 was less effective in M_2_M_3_^**−/−**^ mice than in WT and β_1_β_2_^**−/−**^ mice (Fig. 4c).

To clarify the effect and mechanism of EA at LI11 on jejunal motility, we used both an antagonist and agonist of adrenoceptors and muscarinic receptors to determine the roles of the sympathetic and parasympathetic pathways in normal rats. We used intravenous administration, and thus, the drugs acted systemically on the entire body rather than on only the target nerves. Therefore, the roles of the target nerves could not be specifically verified [41–46]. Because of this limitation, gene knockout animals were used in our study. These mice have frequently been used in studies of neurobiological mechanisms of acupuncture, and their reliability has been demonstrated in previous related studies [47]. The results of the gene knockout study also fully supported our scientific hypothesis. Notably, in the experiments with different EA intensities (Fig. 5), the rate of change of jejunal motility in the β_1_β_2_^−/−^ mice greatly exceeded that of the WT mice. The former group of mice showed a large amplitude change, from 4 mA to 6 mA stimulation intensity, and the maximum stimulation after entering the plateau region was 2 mA higher than that of the WT animals. We propose that the effect of stimulation at LI11 is primarily due to parasympathetic-M receptors. The mutual antagonistic effects between sympathetic and parasympathetic nerves may explain why the rate of change in beta receptor knockout animals subjected to acupuncture is stronger than that of WT mice.

In this study, we observed that the parasympathetic pathway is involved in EA at LI11, but we did not perform further studies of the sympathetic pathway. No sufficient evidence was found to demonstrate that LI11 stimulation inhibits sympathetic nerve activity, thus representing a limitation of our study that should be addressed in further research. However, our previous work has shown that stimulating ST37 (Shangjuxu) excites parasympathetic nerves, and stimulating ST25 (Tianshu) excites sympathetic nerves [40, 48]. All these results together support the “homotopic and heterotopic acupoints” theory proposed by Zhu Bing et al.

To address the mechanism of the effects of EA stimulation at LI11, we sought to characterize the somatic afferents of EA stimulation by using different stimulation intensities. Somatic afferent nerve fibres are composed of Aα- (group I), Aβ- (group II), Aδ- (group III) and C-fibres (group IV) [37, 49]. The mean stimulation thresholds for inducing firing in mouse Aδ- and C-fibres have been reported to be approximately 2 mA and 3 mA, respectively [37]. Thus, we chose different EA stimulation intensities (1, 2, 4, 6, and 8 mA) to test the responses in three types of mice (β_1_β_2_^**−/−**^, M_2_M_3_^**−/−**^ and WT mice). In normal mice (the WT mice group), EA at LI11 significantly increased jejunal motility at all intensities except 1 mA, and when the intensity exceeded 4 mA, the effect plateaued. These results verify that Aδ-fibres are activated by the stimulation when the intensity is maintained at 2 mA and that 4 mA can active C-fibres. The M_2_M_3_^**−/−**^ group differed from the WT and β_1_β_2_^**−/−**^ groups in that the rate of change remained under 105% for all stimulation intensities. This result suggests that activation of muscarinic receptors plays a key role in the effect of EA at LI11, thus further supporting further our hypothesis.

## Conclusion

In summary, we clearly demonstrated the important role of the parasympathetic pathway in the promotion effect of EA stimulation at LI11 on jejunal motility. The intensity-dependent manner of EA stimuli indicates that Aδ-fibres and C-fibres may participate in the response to EA stimulation from 2 mA to 4 mA. LI11, a heterotopic point, also promotes jejunal motility under EA stimulation, similarly to the points on the hind paw. This study provides further evidence that acupuncture regulates gastrointestinal function via the somatic-autonomic reflex.

## Additional file

Additional file 1: Genetic background of gene knockout mice. (DOCX 195 kb)

## Abbreviations

EA: Electroacupuncture
IBS: Irritable bowel syndrome
MA: Manual acupuncture
