# Supplementary material for: Electroacupuncture at LI11 promotes jejunal motility via the parasympathetic pathway
Source: BMC Complement Altern Med. 2017 Jun 21;17:329. doi: 10.1186/s12906-017-1826-9 (PMC5480127; doi:10.1186/s12906-017-1826-9)
Supplement: Additional file 1: — Genetic background of gene knockout mice. (DOCX 195 kb) [file 12906_2017_1826_MOESM1_ESM.docx]

**Additional file**

**Genetic background of gene knockout mice**

Two kinds of gene knockout mice were contributed in our study, including β_1_β_2_**^-/-^** mice (B6.129X1-β_1_β_2_^tm1Jul/NJU^, J003810; donated by the Jackson laboratory in American), M_2_M_3_**^-/-^** mice (B6.129X1-M_2_M_3_^tm1Jul/NJU^, D0407; introduced from Kumamoto University, Japan), and the wild-type counterparts (WT mice; purchased from Model Animal Research Center of Nanjing University, China). In order to confirm the genetic background of the gene knockout mice, Nanjing Biomedical Research Institute of Nanjing University helped us to test with PCR experiments.

**β_1_β_2_^-/-^ mice**

Double beta1/2 adrenergic receptor knockout mice were created by mating Adrb1 homozygous knockout mice with Adrb2 homozygous knockout mice to generate compound heterozygotes and then the offspring mated to obtain compound homozygotes.

Adrb1 null mice were created using a targeting vector containing a neomycin resistance gene driven by the mouse phosphoglycerate kinase promoter to disrupt most of the Adrb1 coding region (all but a 3' 183 bp). The construct was transfected into 129- derived R1 embryonic stem (ES) cells. Correctly targeted ES cells were injected into C57BL/6J blastocysts. The resulting chimeric male animals were mated to C57BL/6J X DBA/2 F1 hybrids.Adrb2 null mice were created in a similar fashion using a targeting vector again containing a neomycin resistance gene driven by the mouse phosphoglycerate kinase promoter to disrupt Adrb2 such that the end of the fourth transmembrane segment is absent, rendering the receptor nonfunctional. The construct was transfected into 129- derived R1 embryonic stem (ES) cells. Correctly targeted ES cells were injected into CD-1 blastocysts. The resulting chimeric male animals were mated to FVB/N females.

**M_2/3_^-/-^ mice**

M2M3-/- mice were introduced from Kumamoto University, Japan. Homozygous mAChR2-/-mice were crossed with homozygous mAChR3-/-mice. The F1 compound heterozygotes were then intercrossed to generate F2 mice. The mAChR2/3-/- mice were obtained at the expected Mendelian ratio and also were interbred to generate the animals used for the experiments described here. F2 wild-type mice were interbred to obtain control mice (mAChR2/3+/+) with an equivalent genetic background [129/J1 (25%) X 129SvEv (25%) X CF1 (50%)].

**The PCR results of gene knockout mice.**

As shown in the S Figure, (A) Chrm2: WT = 510 bp, Mut = 450 bp. (B) Chrm3: WT = 500 bp, Mut = 350 bp, (C) Adrb1: Mut = 350 bp. (D)Adrb1: WT = 183 bp. (E) Adrb2: Mut = 410 bp. WT = 225 bp. WT, wild-type; P: positive control; N: negative control.

Thess results proved the genetic background of the gene knockout mice we used and the next studies using these mice were believable [1-6].


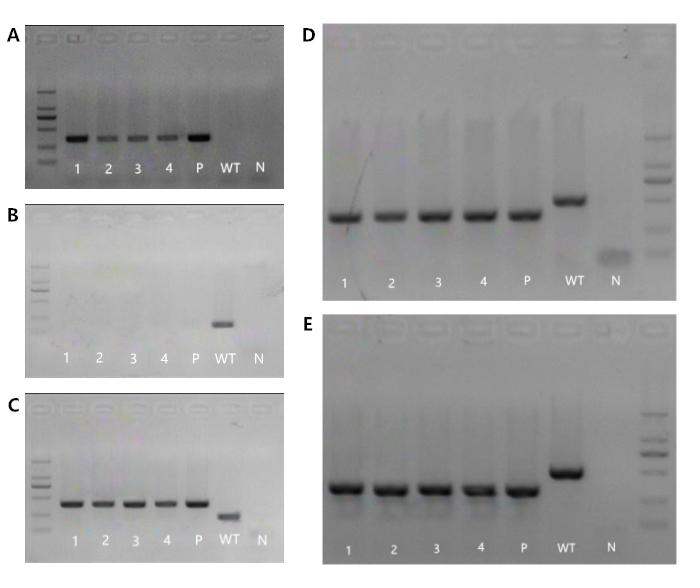


**References**

1. <https://www.jax.org/strain/003810>
2. Gao X, Zhao Y, Su Y, Liu K, Yu X, Cui C, et al.β1/2or M2/3Receptors Are Required for Different Gastrointestinal Motility Responses Induced by Acupuncture at Heterotopic or Homotopic Acupoints. PLoS One. 2016 Dec 15;11(12):e0168200c.
3. Miyakawa T, Yamada M, Duttaroy A, and Wess J. Hyperactivity and intact hippocampus-dependent learning in mice lacking the M1 muscarinic acetylcholine receptor. J Neurosci. 2001; 21:5239–5250.
4. Gomeza J, Shannon H, Kostenis E, Felder C, Zhang L, Brodkin J, Grinberg A, Sheng H, and Wess J. Pronounced pharmacologic deficits in M2 muscarinic acetylcholine receptor knockout mice.Proc Natl Acad Sci. 1999;96:1692–1697.
5. Fisahn A, Yamada M, Duttaroy A, Gan JW, Deng CX, McBain CJ, and Wess J. Muscarinic induction of hippocampal gamma oscillations requires coupling of the M1 receptor to two mixed cation currents. Neuron. 2002;33:615–624.
6. Yamada M, Miyakawa T, Duttaroy A, Yamanaka A, Moriguchi T, Makita R, Ogawa M, Chou CJ, Xia B, Crawley JN, et al. (2001) Mice lacking the M3 muscarinic acetylcholine receptor are hypophagic and lean. Nature. 2001;410:207–212.

**Tables about Differences between Pre-EA and Dur-EA in The Experiments**

When describing the results of our study, we found it could not clearly show the difference of the jejunal pressure between before EA and during EA. As a result, it could not visually display the effect of EA stimulation induced. In order to demonstrate our experimental results in a more comprehensive way, the tables below were made. Results in these tables were expressed as mean ± SEM (standard error of the mean). Data were analyzed using SPSS 19.0 (IBM, Armonk, NY, USA). Paired-sample t tests (within each group) were used for statistical analyses. P < 0.05 was considered statistically significant.

***With or without the the beta adrenoceptor agonist*** ***clenbuterol and antagonist*** ***propranolol in rats***

|  | N | pre-EA | dur-EA | *P* |
| --- | --- | --- | --- | --- |
|  |  | (kPa) | (kPa) |  |
| control | 8 | 0.3223±0.0079 | 0.3492±0.01196 | 0.003^*^ |
| Prop | 8 | 0.4994±0.02267 | 0.53±0.02422 | 0.029^*^ |
| Clen | 8 | 0.1854±0.01626 | 0.1973±0.01798 | 0.003^*^ |

Data are expressed as mean ± SEM, ^*^*P* < 0.05, vs pre-EA, paired t-test. Propranolol promoted jejunal pressure significantly and clenbuterol led to the opposite effect, whereas EA at LI11 increased jejunal pressure in all three groups, which had statistical significance.

***β_1_β_2_*** ***adrenoceptor double-knockout mice and the wild-type counterparts***

|  | N | pre-EA | dur-EA | *P* |
| --- | --- | --- | --- | --- |
|  |  | (kPa) | (kPa) |  |
| WT | 8 | 0.2313±0.00934 | 0.2463±0.01149 | 0.026^*^ |
| β_1_β_2_^-/-^ | 8 | 0.2613±0.00811 | 0.2838±0.01034 | 0.005^*^ |

Data are expressed as mean ± SEM, ^*^*P* < 0.05, vs pre-EA, paired t-test. EA at LI11 increased jejunal pressure of both groups of mice significantly.

***With or without the the muscarinic receptor agonist ACh and antagonist atropine in rats***

|  | N | pre-EA | dur-EA | *P* |
| --- | --- | --- | --- | --- |
|  |  | (kPa) | (kPa) |  |
| control | 8 | 0.3341±0.00818 | 0.3612±0.01238 | 0.003^*^ |
| Ach | 8 | 0.4970±0.02365 | 0.5210±0.02470 | 0.000^*^ |
| Atropine | 8 | 0.2149±0.01489 | 0.2451±0.01536 | 0.072^△^ |

Data are expressed as mean ± SEM, ^*^*P* < 0.05, ^△^*P* > 0.05, vs pre-EA, paired t-test. Compared with the jejunal pressure before EA, changes were observed during EA in the control group and ACh group, but EA at LI11 failed to enhance jejunal pressure in the atropine group.

***With or without vagotomy in rats***

|  | N | pre-EA | dur-EA | *P* |
| --- | --- | --- | --- | --- |
|  |  | (kPa) | (kPa) |  |
| control | 8 | 0.3027±0.00741 | 0.3277±0.01118 | 0.003^*^ |
| sham control | 8 | 0.2293±0.00933 | 0.2442±0.01057 | 0.003^*^ |
| vagotomy | 8 | 0.2248±0.00511 | 0.2271±0.00441 | 0.393^△^ |

Data are expressed as mean ± SEM, ^*^*P* < 0.05, ^△^*P* > 0.05, vs pre-EA, paired t-test. Compared with the jejunal pressure before EA, changes were observed during EA in the control group and the sham control group, but EA at LI11 failed to enhance jejunal pressure in the vagotomy group.

***M_2_M_3_ adrenoceptor double-knockout mice and the wild-type counterparts***

|  | N | pre-EA | dur-EA | *P* |
| --- | --- | --- | --- | --- |
|  |  | (kPa) | (kPa) |  |
| WT | 8 | 0.2306±0.00938 | 0.2475±0.01034 | 0.006^*^ |
| M_2_M_3_^-/-^ | 8 | 0.2569±0.01065 | 0.26±0.01118 | 0.544^△^ |

Data are expressed as mean ± SEM, ^*^*P* < 0.05, ^△^*P* > 0.05, vs pre-EA, paired t-test. Different from WT group, EA at LI11 failed to increase jejunal pressure in M_2_M_3_ adrenoceptor double-knockout mice.
